# Prevalence and histopathological analysis of cystic echinococcosis in ruminants of District Narowal, Pakistan: focus on pulmonary involvement

**DOI:** 10.1590/S1984-29612024080

**Published:** 2024-12-20

**Authors:** Muhammad Usman, Hafiz Muhammad Rizwan, Muhammad Sohail Sajid, Razia Kausar, Urfa Bin Tahir, Haider Abbas, Muhammad Khalil Ateeq, Mohsin Raza, Mahvish Maqbool, Dalia Fouad, Farid S. Ataya

**Affiliations:** 1 Section of Histology, Department of Basic Sciences, KBCMA College of Veterinary and Animal Sciences, Narowal, Sub-campus UVAS, Lahore, Pakistan; 2 Section of Parasitology, Department of Pathobiology, KBCMA College of Veterinary and Animal Sciences, Narowal, Sub-campus UVAS, Lahore, Pakistan; 3 Department of Parasitology, Faculty of Veterinary Sciences, University of Agriculture, Faisalabad, Pakistan; 4 Department of Anatomy, Faculty of Veterinary Science, University of Agriculture, Faisalabad, Pakistan; 5 Section of Physiology, Department of Basic Sciences, KBCMA College of Veterinary and Animal Sciences, Narowal, Sub-campus UVAS, Lahore, Pakistan; 6 Eastwood Lab, Department of Entomology, Virginia Tech University, Blacksburg, USA; 7 Department of Zoology, College of Science, King Saud University, Riyadh, Saudi Arabia; 8 Department of Biochemistry, College of Science, King Saud University, Riyadh, Saudi Arabia

**Keywords:** Epidemiology, hydatid cyst, histopathology, livestock, Narowal, Epidemiologia, cisto hidático, histopatologia, pecuária, Narowal

## Abstract

A total of 384 animals (sheep, goat, cattle, and buffalo) were examined for the presence of hydatid cysts only in the lungs. The lung tissue samples associated with the hydatid cyst were collected immediately after slaughter, followed by fixation in 10% formalin. The fixed tissue was subjected to paraffin embedding technique. Tissue sections of 5 microns were cut by microtome and stained using Harri’s Haematoxilin and Eosin method. Overall, 13.80% of ruminants were found positive for lung infections with hydatid cyst. Only the sex of ruminants showed significant (P < 0.05) association with the infection of hydatid cyst in lungs. All other variables, such as species of ruminants, age, and months showed non-significant (P > 0.05) association. Pulmonary sections taken from infected animals revealed laminated membranes encased in a region with significant (P < 0.05) cellular infiltration (53.4 ± 7.9 µm^2^), primarily composed of lymphocytes, plasma cells, macrophages, and occasionally neutrophils, and eosinophils. In addition, significant (P < 0.05) epithelial disruption in the bronchioles (0.94 ± 0.05 µm^2^) and alveolar septa were also noticed in sections. These histopathological findings lead to the conclusion that pathological changes occur in the tissues surrounding the cyst as well as in areas more distant from the cyst.

## Resumo

Um total de 384 animais (ovinos, caprinos, bovinos e bubalinos) foram examinados para a presença de cistos hidáticos. As amostras de tecido pulmonar associadas aos cistos hidáticos foram coletadas imediatamente após o abate, seguidas pela fixação em formalina a 10%. O tecido fixado foi submetido à técnica de inclusão em parafina. Fragmentos de tecido de 5 micrômetros foram cortadas por micrótomo e coradas, utilizando-se o método de Hematoxilina de Harris e Eosina. No total, 13,80% dos ruminantes foram encontrados positivos para infecções pulmonares com cistos hidáticos. Apenas o sexo dos ruminantes mostrou associação significativa (P < 0,05) com a infecção por cistos hidáticos nos pulmões. Todas as outras variáveis, como a espécie dos ruminantes, idade e meses, mostraram associação não significativa (P > 0,05). Os fragmentos pulmonares retiradas de animais infectados revelaram membranas laminadas, envolvidas em uma região com infiltração celular significativa (P < 0,05) (53,4 ± 7,9 µm^2^), compostas principalmente por linfócitos, plasmócitos, macrófagos e, ocasionalmente, neutrófilos e eosinófilos. Além disso, observou-se significativa (P < 0,05) ruptura epitelial dos bronquíolos (0,94 ± 0,05 µm^2^) e septos alveolares nos fragmentos. Esses achados histopatológicos levam à conclusão de que ocorrem alterações patológicas nos tecidos circundantes ao cisto, bem como em suas áreas mais distantes.

## Introduction

Hydatid disease, also known as cystic echinococcosis (CE), is a significant zoonotic parasitic disease caused by the larval stages of the *Echinococcus* tapeworm especially *E. granulosus*. Other species like *E. multilocularis* are responsible to cause alveolar echinococcosis (Gessese, 2020). In livestock-dependent regions like Pakistan, it carries significant public health and economic implications (Mehmood et al., 2020). Canines are the primary carriers of this parasite, while ruminants, such as sheep, goats, buffalo, and cattle, serve as intermediate hosts (Gottstein et al., 2001). The hydatid cyst is the product of larval infection, where the larvae (called oncospheres) form these cysts, which contain multiple protoscolices. These cysts can develop in different organs of ruminants, with the liver and lungs being very common. However, pulmonary CE, where cysts develop in the lungs, is of particular interest due to its clinical expression and potential serious morbidity in affected animals (Otero-Abad & Torgerson, 2013).

Cystic echinococcosis (also known as hydatidosis) is not just an economic concern due to its effects on livestock production but also poses a public health threat as human exposure can occur through accidental ingestion of *Echinococcus* eggs (Grosso et al., 2012). Ruminants with hydatid cysts in their lungs have severe breathing problems, such as reduced lung capacity, respiratory distress, chronic coughing, and increased susceptibility to secondary infections, which result in deteriorating overall health and productivity (Kumar et al., 2016). In this regard, understanding the prevalence rate and pathological alterations linked with CE is crucial for developing control strategies that effectively counter the zoonotic transmission of such diseases.

Earlier studies have shown that CE is endemic in many parts of Pakistan, particularly in livestock-rearing zones (Haleem et al., 2018; Mehmood et al., 2020; Saleem et al., 2023). It has been noted that liver and lungs are highly susceptible to the disease among ruminants, with various studies reporting varied rates depending on geographical location and management procedures (Abo-Aziza et al., 2019). However, there is no specific information regarding the prevalence and histopathology of pulmonary CE in ruminants within the Narowal district. Most previous studies focused on other areas or did not give detailed information about lung pathology in relation to the disease. The present study hypothesizes that CE of lungs is prevalent among small and large ruminants in Narowal district, and the infection leading to significant histopathological changes in affected animals. This study aimed to determine the prevalence of only CE of lungs and perform a histopathological analysis in sheep, goats, cattle, and buffalo from Narowal district, Pakistan.

The results of this study bridge a significant knowledge gap concerning the prevalence and pathology of pulmonary CE in the study area. Elaborating more on lung tissues through histopathological examinations will give new insights into severity and character of pulmonary involvement in CE, leading to improved diagnostic, therapeutic, and preventive measures. Additionally, knowledge about the local epidemiology of the disease would assist in formulating area-specific strategies for controlling as well as eliminating CE, thus improving animal and public health.

## Materials and Methods

### Study area and study design

**Figure 1:**
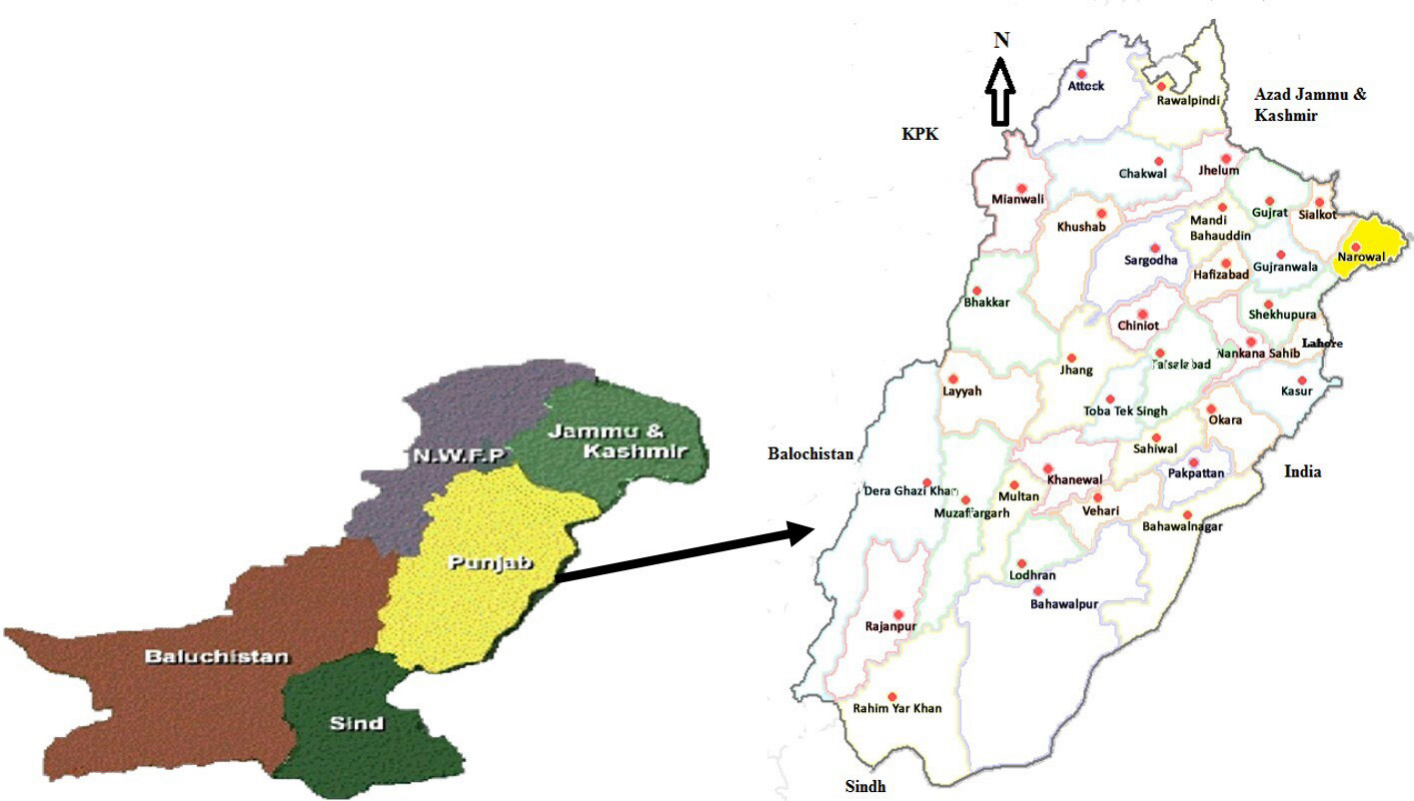
Map of Pakistan and Punjab province highlighting the study District Narowal in yellow.

Narowal city lies on the western bank of the river Ravi in the Punjab province, northeast side of Pakistan (Figure 1), between latitudes 31°55’ to 32°30’ north and longitudes 74°35’ to 75°21’ east (Wikipedia). Narowal district shares its border with Sialkot to the west, Sheikhupura to the south, Jammu and Kashmir to the northwards, and Gurdaspur (India) to the east. The lung samples were collected from both small (sheep and goat) and large (cattle and buffalo) ruminants. A standard formula for simple random sampling was used to calculate the sample size for screening of hydatid cyst infection, assuming an expected prevalence of 50% (Thrusfield et al., 2018). By substituting the values into the formula, a sample size of 384 was calculated (92 sheep, 117 goats, 111 cattle, and 64 buffalo), ensuring a 95% confidence interval and 5% absolute precision (Equation 1).


$$\frac{n=1.96^{2}P_{\exp}\left(1-P_{\exp}\right)}{d^{2}}$$ (1)

where: n = Sample size; *P_exp_ =* Expected prevalence; d^2^ = Desired precision.

Animal variables sex (male and female), age and species as well as months (January to June 2024) of sampling were recorded. In this study, sheep under 9 months and goats under 12 months were classified as young, while older animals were considered adults. Cattle and buffalo were categorized as young if under 24 months and adults if older. Age was determined using dental formula analysis and information from butchers and farmers. Lung tissue samples were collected from both hydatids cyst-infected and non-infected animals to conduct a comparative histopathological analysis (Tehrani et al., 2015). The Molecular Parasitology Laboratory, Faculty of Veterinary Science Research Ethics Committee of University of Agriculture Faisalabad, Pakistan vide letter No. MPL 524/11-23 has approved the current protocol.

### Tissue collection

The animals slaughtered at the local abattoir of Narowal district from January to June 2024 were examined for hydatid cyst infection. The lungs of all animals were carefully checked for hydatid cysts. To collect lung tissues from animals for histopathological comparisons, tissue sections approximately 1 cm^2^ in size were excised aseptically from hydatid cyst-positive and hydatid cyst-negative animals. For infected animals, samples were taken from areas containing visible cysts to capture the local histopathological effects. Non-infected tissues were collected from a region within the lung but without visible cysts to ensure consistency in sample site and tissue characteristics (Zitnan et al., 2008). The collected samples were then immediately fixed in neutral buffered formalin. They were dehydrated using progressively stronger alcohol solutions, cleaned with xylene, penetrated by melted paraffin wax, and embedded using an embedding desk. Tissue blocks were sliced into 5 µm thick sections using a microtome and placed onto glass slides. H&E staining was done on tissue slides (Suvarna et al., 2019), which were then examined under a microscope (B-150, Optika, Italy) for any histological changes at 40X and 100X magnification.

### Microscopic examination of tissue

Photomicrographs of the stained sections of lung tissues obtained under various magnifications were also analyzed using ImageJ software (Softonic, 2024) to measure histological features, such as disruption of typical respiratory epithelium (TRE), infiltration of inflammatory cells, and septa health (Beigh et al., 2017).

### Statistical analyses

To determine the association of sex, age, months of sampling, and ruminant species with the hydatid cyst infection, the Chi-square test was employed. A completely randomized design was used to analyze the histological features data. One-way ANOVA was used to compare means and standard errors, where significant differences were determined using Tukey’s test to compare the means of histopathological parameters. Data were analyzed using Minitab 17.0 software (Minitab Inc., Pennsylvania, USA). Any P value equal to or below 0.05 was considered statistically significant. We calculated the relative prevalence (%) for hydatid cyst infection by dividing the number of positive samples by the total number tested and multiplying by 100.

## Results

**Figure 2:**
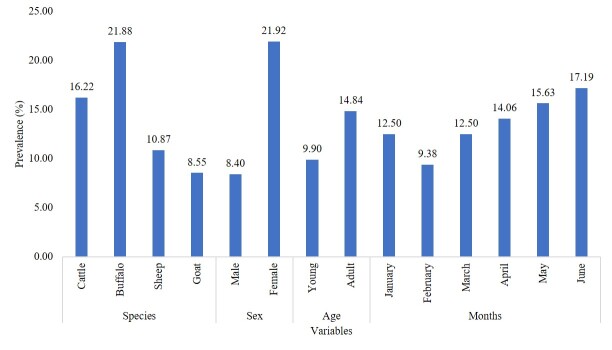
Prevalence of pulmonary cystic echinococcosis infection in ruminants of district Narowal, Punjab, Pakistan.

The overall prevalence of pulmonary CE infection in the ruminants of district Narowal, Punjab, Pakistan was 13.80%. Among different species of ruminants, the highest prevalence of pulmonary CE infection was found in buffalo (21.88%) followed by cattle (16.22%), sheep (10.87%), and goat (8.55%). The association between pulmonary CE infection and different species of ruminants was non-significant (P > 0.05). However, sex showed a significant (P < 0.05) association with pulmonary CE infection, with the highest prevalence in females (21.92%). Age and months showed a non-significant (P > 0.05) association with pulmonary CE infection. Overall, adult animals showed the highest infection rates as compared to young animals. Among months, the highest prevalence was recorded during June (17.19%) and the lowest during February (9.38%). The frequency distribution of pulmonary CE infection is given in Table 1, and the prevalence of pulmonary CE infection among different variables is given in Figure 2.

**Table 1:** Frequency distribution of hydatid cyst infection in ruminants of district Narowal, Punjab, Pakistan.

**Character**	**Variables**	**Examined**	**Positive**	**Prevalence (% ± C.I.)**	**X^2^ value**	**P-value**
Species	Cattle	111	18	16.22 ± 6.86	5.621	0.132
	Buffalo	64	14	21.88 ± 10.13		
	Sheep	92	10	10.87 ± 6.36		
	Goat	117	10	8.55 ± 5.07		
Sex	Male	238	20	8.40 ± 4.52	10.485	0.001^*^
	Female	146	32	21.92 ± 6.71		
Age	Young	101	10	9.90 ± 5.82	1.207	0.272
	Adult	283	42	14.84 ± 4.14		
Months	January	64	8	12.50 ± 8.10	1.566	0.905
	February	64	6	9.38 ± 7.15		
	March	64	8	12.50 ± 8.10		
	April	64	9	14.06 ± 8.51		
	May	64	10	15.63 ± 8.90		
	June	64	11	17.19 ± 9.25		

Abbreviations: C.I.: 95% confidence interval.

* Statistically significant, p < 0.05.

**Figure 3:**
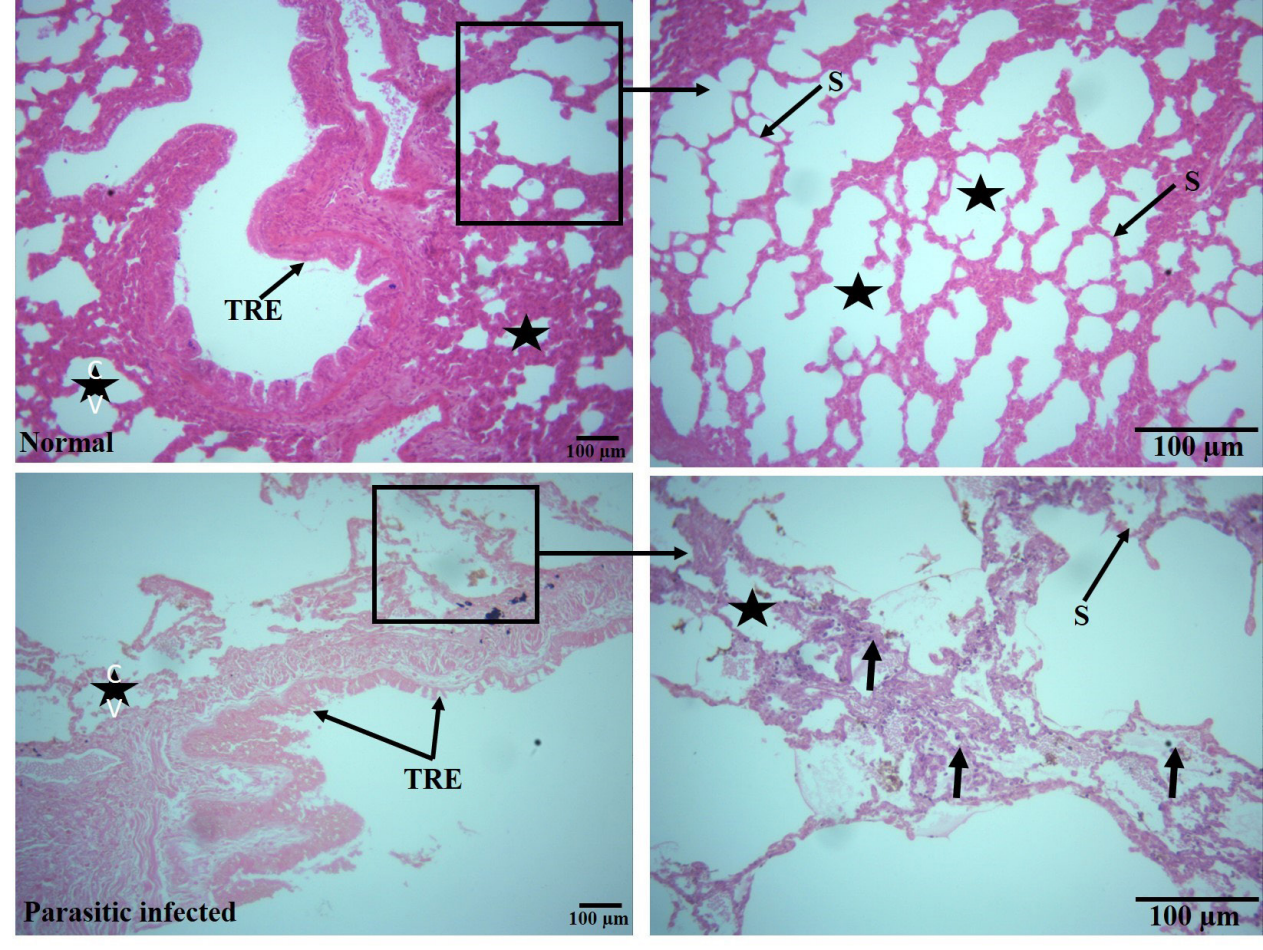
Histo-micrograph of lung tissue taken from normal group (upper row) and parasitic infected group (lower row). In normal groups, the tissue sections showed normal contents of lungs histological architecture. A respiratory bronchiole with Typical respiratory epithelium (TRE) can be seen with normal interalveolar septa (S) and alveolar sac (black star) which form more than 80% of normal lungs. On the contrary, the histological sections of parasitic infected groups vary greatly as compared to normal one. The TRE was found disrupted and its thickness decreased significantly. The interalveolar septa (S) and alveolar sacs were found severely damaged and infiltrations of inflammatory cells were also observed there (small arrow) (H&E, first column 40X, second column 100X, scale bar 100 µm).

**Figure 4:**
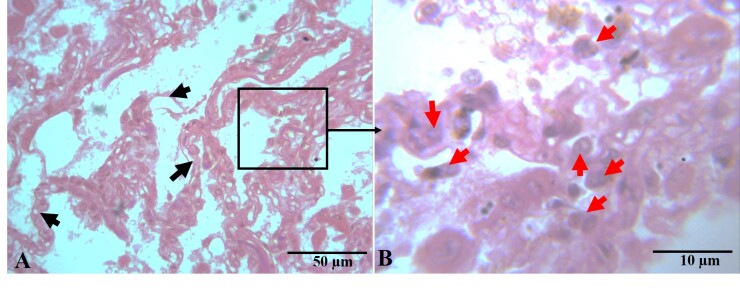
Histo-micrograph of parasitic infected lung section. Pulmonary tissue presented on the right side picture (A) exhibited the damaged air sac septa epithelium (black arrow head) and (B) excessive infiltration of inflammatory cells (red arrow head) were observed in the left side picture (H & E, A: 400X, scale bar 50µm; B: 1000X, scale bar 10µm).

Cellular architecture of the lungs was also found to be significantly affected by parasitic infection. The histological structure of the lungs, including alveolar septa, alveolar sacs, and respiratory bronchioles, which comprise more than 80% of the lung’s architecture, was severely damaged and altered, with congested pulmonary veins indicating decreased efficiency of gaseous exchange in the lungs. Additionally, the typical respiratory epithelium of the respiratory bronchiole was ruptured and found to be thinner (0.94 ± 0.05 µm) compared to the normal thickness (1.65 ± 0.24 µm). The area of cellular infiltration (µm^2^) by inflammatory cells also increased significantly in the parasitic-infected group (53.40 ± 7.0), while in normal lungs, this value was 12.17 ± 0.27. The cellular infiltration primarily comprised lymphocytes, plasma cells, macrophages, and occasionally neutrophils and eosinophils (Table 2, Figure 3). Histo-micrograph of lungs showing infiltration of inflammatory cells with severe septal damage is given on Figure 4.

**Table 2:** Statistical values (Mean ± SEM) of different histological aspect of lung tissues of normal and infected groups of ruminants.

**Variables**	**Normal**	**Parasitic infected**
Epithelium Thickness (**µm**)	1.65 ± 0.24 a	0.94 ± 0.05b
Cellular infiltration (%)	12.17 ± 0.27 b	53.40 ± 7.0 a

Means sharing different superscript a and b in a row are statistically different at P < 0.05.

## Discussion

The presence of CE in livestock has high epidemiological significance as it gives an impression on how a high risk of transmission to other humans and animals within the endemic zone exists (Saleem et al., 2023). In the present study, the overall prevalence rates observed among different livestock species indicate a sustained existence of *Echinococcus* in the district, underscoring the need for continuous surveillance and control measures. The CE is a zoonotic disease, and when animals are found with high prevalence, public health risk is also high especially in areas with interactions between livestock and humans and poor sanitation (Haleem et al., 2018). Differences in prevalence between species and age groups also suggest other factors that increased infection risks such as potential grazing habits and exposure of animals to contaminated environments (Torgerson et al., 2010). To reduce the socio-economic impact associated with CE, it is important to understand these processes in order to make specific therapies aimed at reducing the number of cases of CE in both humans and animals (Haleem et al., 2018; Widdicombe et al., 2022). Moreover, coverage of a seasonally averted prevalence pattern, which is unexpected in CE, can be useful in explaining regional determinants of parasite transmission, indicating the need for regional epidemiology.

The study showed variations in the prevalence of hydatid cyst infections among different ruminant species, genders, and age groups as well as across months. Lower prevalence rates have been observed in this study compared to some parts of Ethiopia and Iran, where certain ruminant populations reported prevalence rates as high as 30-40% (Berhe, 2009; Torgerson et al., 2010). This could be due to differences in environmental factors, livestock management practices, and the presence of definitive hosts (canines) in various areas. For example, the *Echinococcus* transmission cycle is likely more intense in regions with a higher density of stray dogs or traditional pastoralist practices, leading to higher prevalence rates (Macpherson, 2005). Dogs act as definitive hosts of *Echinococcus*, and acquired infection by ingestion of contaminated visceral organs of animals. In many rural and slaughterhouse communities, organs infected with CE are not discarded properly but are through around the slaughtering facility or offered to dogs as cultural practices. Such kind of practices can increase the risk of transmission, as infected dogs release tapeworm eggs through faeces into the environment, and contaminate the soil, vegetation, and water, and maintain the disease cycle. Proper disposal of infected organs is important to control the spread of the CE.

Among the species, buffaloes had the highest prevalence (21.88%), followed by cattle (16.22%), sheep (10.87%), and goats (8.55%). The present findings on species-related differences in hydatid cyst infection are similar to those found elsewhere. For example, a study conducted in Ethiopia indicated that buffalo had a higher rate of infection than other ruminants (Kebede et al., 2009). This has been attributed to different grazing habits that expose them to canine feces and different management practices. Buffalos, mostly reared under open grazing systems, are more likely to come into contact with contaminated pastures, which may explain the higher prevalence observed during this investigation.

Females were significantly associated with a higher proportion of hydatid cysts compared with male ruminants. In line with this finding, previous studies also demonstrated that females were at an increased risk of being affected by CE when compared with males (Getaw et al., 2010; Saleem et al., 2023). This difference could possibly be attributed to the fact that female animals are usually kept for breeding purposes, thus spending more time exposed to the parasite compared to males, which are often sold or slaughtered when young (Vieira et al., 2014). The study also found that adult animals had a higher prevalence of CE (14.84%) than younger ones (9.90%), although this was not statistically significant (P = 0.272). Similar findings have been reported in other studies like those by Ibrahim (2010) and Romig et al. (2006), where adult ruminants had higher infection rates. This could be due to older animals being cumulatively exposed to the parasite over time, increasing their chances of contracting it.

Surprisingly, there were no significant differences in the rate of occurrence of hydatid cysts between the months under investigation (P = 0.905). Nonetheless, a slight increase from January (12.50%) to June (17.19%) was observed, which may be attributed to changes in grazing patterns, environmental factors, and the activity of intermediate hosts, as stated in studies conducted elsewhere (Dalimi et al., 2002). However, the lack of a monthly effect suggests relative stability throughout the year in the transmission dynamics of CE in Narowal district as opposed to other regions with more pronounced seasonal variations in parasite transmission (Eckert & Deplazes, 2004).

The histopathological examination of lung tissues showed important differences between normal and parasite-infected groups. Specifically, the thickness of the respiratory epithelium (TRE) was significantly decreased in infected lungs (0.94 ± 0.05 µm) as compared to normal ones (1.65 ± 0.24 µm). This thinning of the epithelium could be a direct result of mechanical pressure exerted by growing hydatid cysts, leading to atrophy and compression of the surrounding lung tissues. Similar pathological changes have been previously reported in the lungs of hydatid-infected animals (Daryani et al., 2007).

Furthermore, there was a marked increase in cellular infiltration in the infected lungs (53.40 ± 7.0%) compared to normal lungs (12.17 ± 0.27%). The high level of cellular infiltration, which consisted primarily of inflammatory cells, indicates an intense immune response against the parasitic infection. This inflammatory response may lead to more tissue destruction with possible clinical manifestations like those observed in cases of respiratory distress among affected animals. Similar inflammatory responses have also been reported by Jenkins et al. (2005) in hydatid-infected tissues, supporting our findings. Histopathological examination of lung sections revealed fibrous tissue reactions (capsules), necrosis, cellular reactions, and collapsed lung tissue adjacent to the cyst wall which are in line with Al-Bermani & AlـDabhawi (2022). Additionally, vascular congestion and multiple foci of hemorrhage, particularly in alveolar capillaries were also observed. These hemorrhages are likely due to mechanical pressure exerted by the expanding hydatid cysts of infected animals (Alsulami, 2019; Moudgil et al., 2019).

## Conclusions

Findings from this study highlight that Narowal district experiences a significant burden due to CE, thereby having serious implications for both human and animal health as well as disease control programs in the area. Higher prevalence rates among buffaloes and female ruminants imply that targeting interventions in these groups could be particularly effective in reducing the overall disease burden. Furthermore, histopathological insights into lung involvement provide important knowledge about how the condition affects the respiratory system, which is crucial for strategic development and better management of afflicted animals. Given that CE is a zoonotic disease this study emphasized a one health approach integrating human-environmental model, which are necessary for controlling the disease. Further studies should identify specific risk factors contributing to the varying levels of prevalence observed in different species and regions. The economic impacts of this disease on livestock production also need to be studied, as well as the efficacy of various interventions in reducing the transmission of *Echinococcus*.
